# Evaluability assessment of a targeted national anti-child poverty policy: option appraisal for evaluating the Scottish Child Payment

**DOI:** 10.1186/s12961-026-01489-y

**Published:** 2026-06-17

**Authors:** Michal Shimonovich, Olivia K. L. Hamilton, Peter Craig, Gerard McCartney, Anna Pearce, S. Vittal Katikireddi, Rachel M. Thomson

**Affiliations:** 1 School of Health and Wellbeing, University of Glasgow, 90 Byres Road, Glasgow, G12 8TB United Kingdom; 2 School of Social and Political Sciences, University of Glasgow, Adam Smith Building, Bute Gardens, Glasgow, G12 8RT United Kingdom

**Keywords:** Evaluability assessment, Evaluation planning, Child poverty, Cash transfer

## Abstract

**Background:**

The Scottish Child Payment (SCP) is a recurring cash transfer introduced in 2021 for low-income families, unique within the United Kingdom and forming a core part of the Scottish Government’s statutory child poverty reduction strategy. As a long-term, population-level intervention, the SCP presents an important opportunity to generate evidence on the impact of cash transfers. However, multiple policy changes have made evaluation design challenging. We conducted an evaluability assessment (EA) to identify feasible, policy-relevant evaluation options.

**Methods:**

Our five-stage EA approach included: (1) EA inception, (2) developing a theory of change, (3) prioritizing outcomes and identifying available data, (4) developing evaluation designs and (5) appraisal and reporting. Two stakeholder workshops, informed by evidence reviews, were held. The first refined the Scottish Government’s theory of change. The second focussed on identifying priority outcomes, available data sources and suitable evaluation designs. Stakeholders reviewed a final report summarizing these options.

**Results:**

Given extensive existing and planned qualitative research, stakeholders agreed that a quantitative evaluation should be given precedence. They agreed that child development and child and maternal health and wellbeing outcomes should be prioritized and measured using administrative data. Due to data access constraints, SCP exposure would be estimated using eligibility indicators rather than direct receipt. Evaluating effects on health inequalities for Scottish Government-identified priority family groups compared with the rest of the population was considered important, though linkage of household-level benefits data remains limited. We identified four feasible natural experimental designs: before-and-after analysis, interrupted time series (ITS), controlled ITS and regression discontinuity design (RDD). Each design may be suited to different questions, populations and outcome types, with multiple designs likely to be helpful.

**Conclusions:**

This EA demonstrates that a robust quantitative evaluation of the SCP is both valuable and feasible, but must be tailored to specific outcomes, implementation phases and evolving policy contexts. Linked administrative data offer strong potential, though advocating for more precise measures of SCP receipt and household socioeconomic context to explore inequalities should continue. Estimating the SCP’s effect on child and maternal health should be a priority to inform the design of future poverty reduction policies.

**Supplementary Information:**

The online version contains supplementary material available at https://doi.org/10.1186/s12961-026-01489-y.

## Introduction

Poverty is a major factor influencing health, impacting the most disadvantaged groups and driving health inequalities [1, 2]. Children in poverty face higher risks of low birthweight [3], injuries [4] and developmental issues [5], whilst their parents often struggle with health-related behaviours such as smoking [6], breastfeeding and immunization [7]. Tackling these early life disadvantages, the impacts of which persist into adulthood and across generations, is crucial to promoting lifelong health for all [8]. The severe health consequences of child poverty are particularly concerning in light of a recent United Nations Children’s Fund (UNICEF) report showing a 20% increase in child poverty in the United Kingdom between 2014 and 2021 – the largest rise amongst the 40 high- and upper-middle-income countries examined [9–11]. The UK Government has historically taken primary responsibility for tackling child poverty, which currently affects a third of children when defined as living in households earning 60% or less of the median income (after housing costs) [12]. In the context of austerity and increasing poverty, devolved governments have pursued their own poverty reduction solutions using new powers to create social security benefits [13, 14].

In Scotland, the devolution of social security was established by the Scotland Act 2016, whilst the power to top-up United-Kingdom-wide benefits was granted by the Social Security Act 2018 [14]. These legislative changes enabled the Scottish Government to introduce the Scottish Child Payment (SCP), a cash transfer for low-income families first rolled out in February 2021 [15, 16]. Though many families report that the SCP has been vital for financial stability, particularly during the ongoing cost-of-living crisis, its broader impact remains unclear [17–19]. With annual spending on the SCP projected to reach £492 million by 2028/29, there is a great interest in examining its population-wide impacts, as well as for the six priority family groups that the Scottish Government has identified as being at higher risk of poverty [20, 21]. These groups are: minority ethnic families, lone parent families, families with a disabled person, families with three or more children, families with children under 1 year and families with mothers aged 25 years or younger [22, 23].

The SCP is unique within the United Kingdom, and a full-scale evaluation of its potential co-benefits would not only inform future decisions around the SCP, but also help local and devolved governments plan their own poverty reduction programmes [24, 25]. As a national programme currently benefitting more than 330,000 people each year, an evaluation of the SCP has the potential to strengthen the understanding of the broader, population-wide impacts of cash transfer policies [1, 11]. Whilst research suggests that cash transfers improve health, many studies are set in low- and middle-income countries [26, 27]. Research in high-income countries is often based on small-scale pilots with limited generalizability [28], or is otherwise focussed on cash transfers that have been recently cut short, making it difficult to assess their-long term effects [29, 30]. Additionally, the impact of cash transfers depends on factors such as the target population, amount, frequency and conditions of the transfer [1, 31, 32]. Evaluating the SCP could provide valuable insights into these factors and enhance understanding of the complex relationship between poverty and health.

The aim of this study is to conduct an evaluability assessment (EA) of the SCP, and through this process, identify potential evaluation design options. An EA is a systematic and collaborative approach to evaluation planning, with researchers and intervention stakeholders systematically exploring the feasibility, practicality and utility of an evaluation [33]. An EA can help ensure future evaluations are useful to stakeholders [34]. Designing evaluations for complex policies such as the SCP requires careful consideration, especially as it has undergone several changes, some of which have been unplanned [35].

**Fig. 1:**
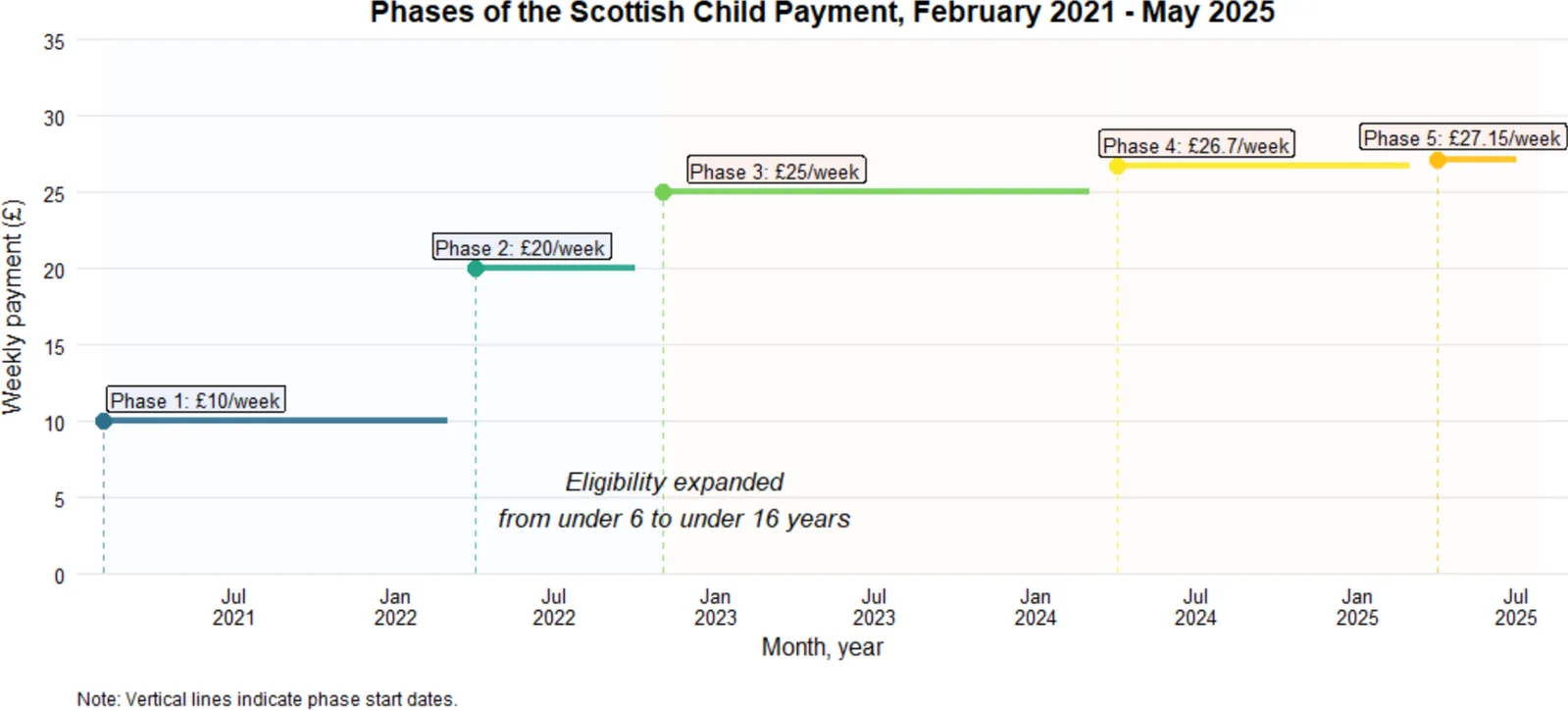
Value and eligibility of each phase of the Scottish Child Payment, by year and quarter (as of May 2025)

There are five key phases marking changes in the value and eligibility criteria for the SCP (see Fig. 1). When first introduced, eligible parents or guardians received £10 per week for each child under 6 years (phase 1). This amount increased to £20 in April 2022 (phase 2), and in November 2022, the payment was raised to £25 per week with updated eligibility criteria including children under 16 years (phase 3) [15]. In its fourth phase (April 2024–March 2025), the SCP provided £26.70 to children under 16 [36]. This increased to £27.15 per child in April 2025, at the start of the fifth and current phase (at the time of writing, May 2025), to keep pace with inflation [37].

## Methods

**Fig. 2:**
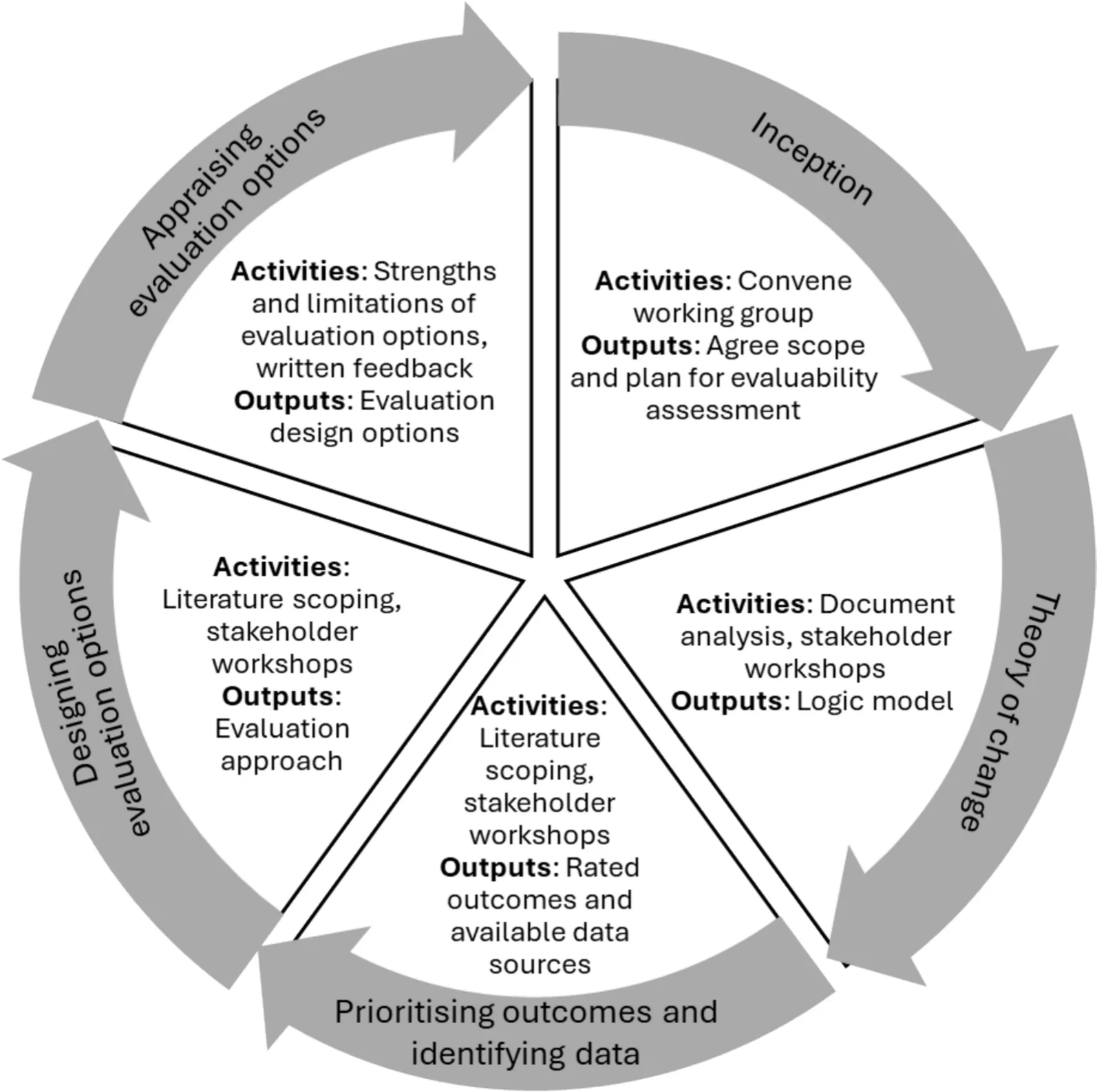
Evaluability assessment stages, activities and outcomes (stages adapted from [39])

We conducted an EA of the SCP following the five-stage approach used for other national interventions with potential for population-level impact [38–40]. These stages are (1) inception of the EA, (2) development of a theory of change, (3) prioritization of outcomes and identification of data requirements, (4) development of evaluation options and (5) appraisal and reporting of these options. The stages are presented linearly, however, in practice, an EA is inherently iterative, with activities such as literature reviews and stakeholder workshops often overlapping across different stages (see Fig. 2) [41].

### Inception

The EA process begins with the inception stage, where planners define the aim and scope of the activities and possible evaluation [42]. This EA was part of a broader NIHR-funded project (NIHR154243) led by the MRC/CSO Social and Public Health Sciences Unit (SPHSU) and conducted in collaboration with Public Health Scotland and the Scottish Government. Public engagement activities as part of the broader project identified income support policies as a key focus, leading the funder, advisory group and SPHSU team to agree on an EA of the SCP. EAs are typically conducted over two to three workshops [34], but two workshops were deemed sufficient due to ongoing Scottish Government research on the SCP, including an interim evaluation that includes a theory of change [43–46]. Workshop 1 focussed on refining the Scottish Government’s theory of change, whilst workshop 2 explored data sources, outcome priorities and evaluation designs. Stakeholders also reviewed a report summarizing the design options. Stakeholders were selected from government departments overseeing SCP administration and public health, poverty charities, think tanks and research agencies (see Supplementary Table 1 for more information about stakeholder organizations). The workshops took place at the SPSHU offices in Glasgow, UK and were jointly conducted with Public Health Scotland with support from the Scottish Government.

### Theory of change

The next EA stage is developing a theory of change, often visualized in a logic model, outlining how the intervention is expected to make a difference [33, 47]. SPSHU researchers incorporated insights from the literature into the Scottish Government’s theory of change, which was further refined in the workshops. Stakeholders assessed the overall plausibility of the SCP achieving its goals and examined its components, which are inputs, activities, outputs, outcomes and context [48, 49]. They were then divided into three breakout groups to have more in-depth discussions, including around the utility of each outcome, potential indicators and expected impacts [47, 49]. Feedback was collected through Padlet and Mentimeter.

### Prioritizing outcomes and understanding data requirements

The third stage of the EA process focussed on the practicality and utility of measuring outcomes given the available data [34, 50]. Given the number of outcomes included in the Scottish Government’s theory of change, this stage was carried out across both workshops. First, stakeholders rated the importance of each outcome on a scale from 1 (least important) to 10 (most important) on the basis of the discussions in the break-out groups. Next, one of the SPSHU researchers (MS) presented potential data sources, drawing from evaluations in Scotland considering similar outcomes [1, 51–53]. Stakeholders assessed the appropriateness of these data sources for measuring the outcomes of interest and rated the feasibility of measuring each outcome on a 1–10 scale, from least to most feasible. The combined importance and feasibility ratings, along with stakeholder insights and existing evidence, highlighted the outcomes considered priorities for future evaluations.

### Developing, reporting and appraising evaluation design options

Stakeholders agreed that a future evaluation should focus on estimating the SCP’s effectiveness using natural experimental methods, discussed further below. To support this, MS presented a high-level overview of potential quantitative study designs. We then explored their strengths and limitations to inform the final EA stage of appraising evaluation design options. Researchers documented these discussions and shared them with stakeholders who provided written feedback.

## Findings

The previous section outlined the activities carried out between November 2023 and April 2024 to explore the evaluability of the SCP. These included stakeholder workshops, reviews of relevant literature and policy documents and a structured process for gathering stakeholder feedback. Together, these activities helped refine a theory of change, establish priorities for future evaluations and appraise potential evaluation design options. The findings presented below reflect the insights gathered during this period.

### Theory of change

**Fig. 3:**
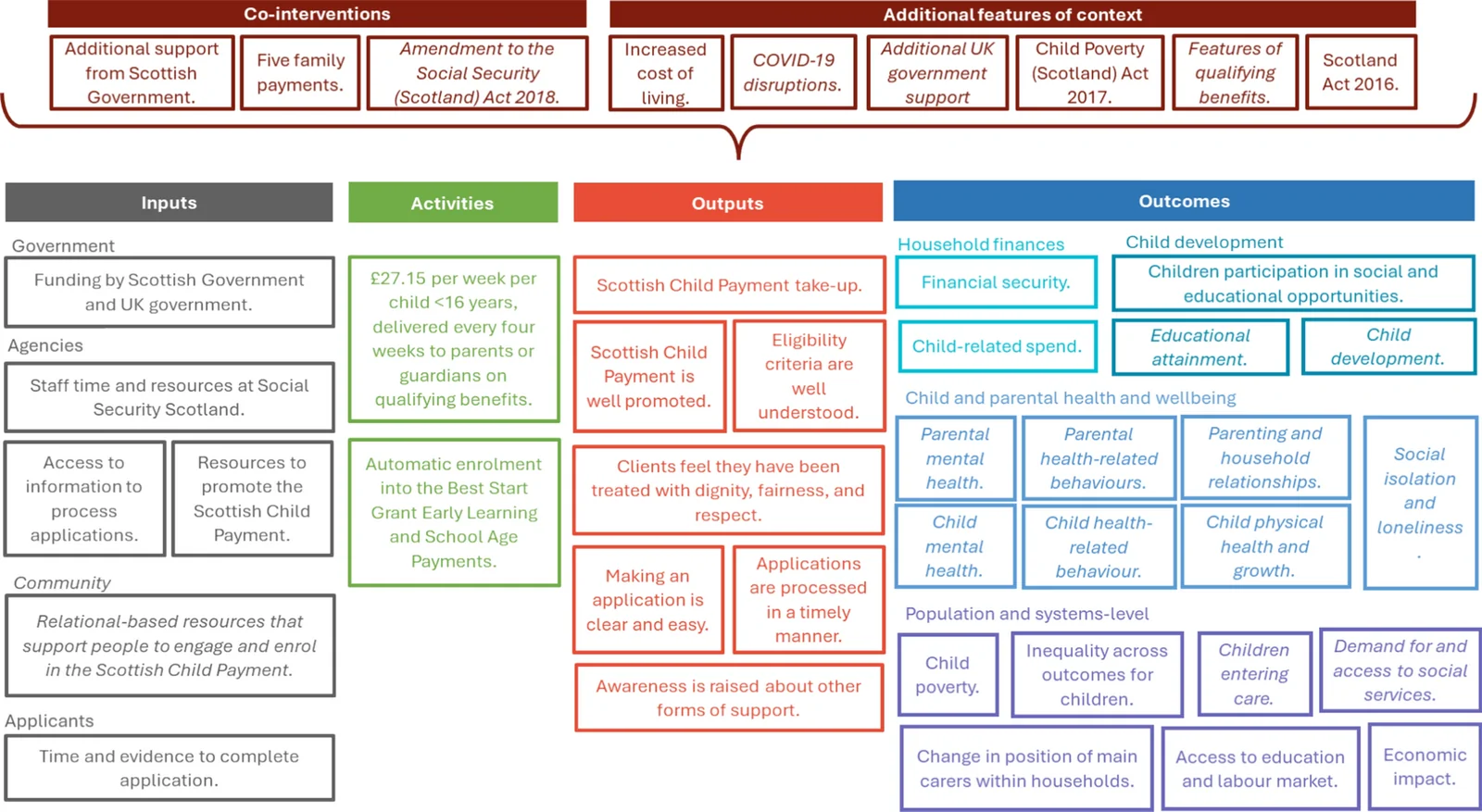
Revised theory of change for the Scottish Child Payment, adapted with stakeholders from Scottish Government’s logic model

Figure 3 presents the revised version of the Scottish Government logic model [44], co-produced with stakeholders and organized by the main theory of change components.

Stakeholders largely agreed with the initial, amended theory of change, resulting in minimal changes to the inputs (the resources required to implement a policy or programme) as well as the activities and outputs that are essential for achieving the desired outcomes (see Supplementary tables 2 and 3 for more detail) [33]. Discussions focussed on factors influencing SCP uptake amongst eligible families, with stakeholders expecting it to rise from 85% at the time of the workshops to around 95%, similar to other benefits [21, 54–56]. Stakeholders identified several key implementation features likely to promote uptake, including capacity for Social Security Scotland staff to review applications, effective promotion, clear eligibility criteria and a straightforward application process (see inputs and outputs section in Fig. 3). They supported the outcomes included in the theory of change but suggested further clarification, including defining and grouping the outcomes thematically (see Table 1). Stakeholders also emphasized the importance of assessing whether the SCP improved the outcomes amongst the six priority family groups, given the risk of inequalities widening between SCP recipients if the benefits are concentrated in the least deprived of the eligible population [22, 23].

**Table 1:** Stakeholder’s descriptions of the outcomes of the Scottish Child Payment (SCP), by outcome domain

Outcomes	Descriptions and example measures
Household finances	
Financial security	The household income necessary for affording basic expenses, creating financial reserves and reducing financial pressure
Child-related spending	Spending on activities, services and goods which are directly relevant to child wellbeing and development
Child development	
Child participation in social and educational opportunities	Social and educational opportunities that children can access
Child development before start of school	Physical, cognitive, emotional and social developmental early years outcomes
Educational attainment	The level of education reached, measured using a variety of indicators (e.g. years of education, grades)
Parent and child health and wellbeing	
Parental mental health	Mental health symptoms, conditions, prescribing or diagnosis of parent(s) or guardian(s)
Parental health-related behaviours	Healthcare use and health-related behaviours of parent(s) or guardian(s) (e.g. immunization, breastfeeding)
Social isolation and loneliness	The level of, and wellbeing measures associated with, social contact and community involvement
Child mental health	Child mental health symptoms, conditions, prescribing or diagnosis
Parenting and household relationships	Intra-household relationships, such as the emotional connection and interaction between parent and child, and parenting style
Child physical health and growth	The key physical health outcomes relevant for healthy growth, such as weight, height, illnesses and unintentional injuries
Child health-related behaviours	The activities relevant for physical health such as physical activity, diet and sedentary behaviour
Population and systems level outcomes	
Child poverty	The proportion of children and young people who are living in poverty (e.g. relative poverty, persistent poverty, deep poverty)
Inequality of outcomes for children	The change in individual or household level outcomes (e.g. educational attainment, child health) between the most and least deprived areas of Scotland
Children entering care	Incidence of rate of children entering care
Demand for social services	Reduced demand for services, including those which provide support for child development (e.g. speech and language therapy) and health (e.g. healthcare)
Access to social services	Barriers and facilitators impacting participation in the key activities, goods and services within their community or society
Change in position of main carers within households	Improved gender equality and female empowerment across a population or community following an improved intra-household position for the main carers to whom SCP is paid (these are predominantly women)
Access to education and labour market	Barriers and facilitators impacting participation in the education and labour market
Economic impact	Impact on Scottish economy such as demand for local labour, goods and services

*SCP* Scottish Child Payment

The primary changes to the proposed theory of change related to context, that is, factors influencing the implementation or success of the SCP that should be considered in the evaluation (see Table 2). Discussions primarily focussed on co-interventions, which are other policies introduced around the same time or after the SCP but are not part of it, such as additional UK Government support during the cost-of-living crisis [57]. One important co-intervention is Best Start, a set of Scotland-specific family benefits that were introduced under the same poverty-reduction legislation as the SCP [58]. Best Start Grants provides one-off payments during three key periods: pregnancy, the early years and as starting school (totalling £1023–£1407 per child, depending on birth order). Best Start Foods is a pre-paid card to help low-income families buy healthy food (paid every 4 weeks, totalling £21.60 to £43.20, depending on the age of the child) [22, 23].

**Table 2:** Description of the co-interventions and key features of context of the Scottish Child Payment

Feature	Description and notes
Co-interventions	
Five Family payments*	Support for low-income families delivered by Social Security Scotland as part of Scottish Government’s poverty reduction efforts. Includes the Scottish Child Payment (SCP), introduced in February 2021. These are (with dates when they were introduced): Best Start Grant Pregnancy and Baby Payment (December 2018): a one-off payment of £754.65 for a first child or £377.35 for a second or later child paid during pregnancy or until the baby is 6 months old Best Start Grant Early Learning Payment (April 2019): a one-off payment of £314.45 per child from 2 to 3.5 years old Best Start Grant School Age Payment (June 2019): a one-off payment of £314.45 per child around school age Best Start Foods Money (August 2019): pre-paid chip and pin card to buy healthy food including eggs, milk, fruit, vegetables (£5.30 weekly during pregnancy; £10.60 weekly from birth until the child’s first birthday; £5.30 weekly for any child from the age of 1 year until the age of 3 years)
Additional government support*	Most of the additional government support provided since the SCP was introduced (beyond the Five Family payments) was intended to address the cost-of-living crisis that began at the end of 2021 [103]. Since this support was devolved, most additional government assistance discussed in the stakeholder workshops was provided by the Scottish Government and delivered through Social Security Scotland, including: Job Start Payment: one-off payment of £314.45 for a young person (ages 16–24 years), or £503.10 for a young person with children to help with the costs associated with the transition into employment. It is available to those who have been out of paid work and receiving a low-income benefit for 6 months prior to finding employment [104] Discretionary Housing Payment: extra discretionary payments made by local authorities to mitigate the effects of the bedroom tax and the benefit cap and to help pay housing costs. Those who receive housing benefit or UC, which includes an amount for housing costs, are entitled to this additional payment [105] Winter Heating Payment (separate from the UK Government’s Winter Fuel Payment): one-off winter payment of (£55.05 in 2023/2024) to help people on low-income benefits with extra heating needs [106] Scottish Welfare Fund: includes the Crisis Grant (to help with an unexpected emergency) and the Community Care Grant (to help with living a settled life in the community). Available to those over the age of 16 years on a low income [107] In January 2022 the Scottish Government extended their free school meals policy to include all pupils in primary 1 (ages 4 or 5 years) to primary 5 (ages 8 or 9 years) at schools run by a local council or funded by the Scottish Government. After primary 5, children of families experiencing financial hardship or on qualifying benefits, or pupils aged 17 or 18 experiencing financial hardship or on qualifying benefits, are also entitled to free school meals [108]
Amendment to the Social Security (Scotland) Act 2018	The SCP is currently delivered under Section 79 of the Social Security (Scotland) Act 2018, which gave the Scottish Government specific authority to top up reserved benefits for people who require additional financial assistance. The SCP is not considered in tax credit or United-Kingdom-wide benefits assessments (i.e. the additional income is “disregarded” by DWP and HMRC [16]). This legislative change provided greater flexibility in delivering the SCP and improved alignment across the Five Family Payments
Additional features of context	
Coronavirus disease 2019 (COVID-19) disruptions	This refers to disruptions resulting from the COVID-19 pandemic and lockdown (e.g. disruptions in employment, learning or community activities)
Increased cost of living	There were increases in the cost of essential goods and services across the United Kingdom in the 12 months to November 2023 [109] (ahead of the first evaluability assessment workshop), including: Average private rental prices increased by 6.2%, which represents the largest annual percentage change since this UK data series began in January 2016 [110] Food and nonalcoholic beverages prices rose by 9.2%. This was down from a high of 19.2% in March 2023, which was the highest annual rate for over 45 years In March 2023, UK annual energy price inflation was the highest amongst G7 economies at around 40.5% [111]
Features of qualifying benefit*	There are several characteristics of the qualifying benefits which are important to the implementation and effect of the SCP, including: Eligibility criteria and method for assessing eligibility criteria (targeting method): Eligibility for universal credit (UC), which is required to be eligible for SCP, is determined by earned income, which is assessed on a 4-weekly basis. The frequency of earned income payments and their overlap with the assessment period can affect eligibility for and cap on UC [112] Conditionality and consequences of nonadherence to conditions: There are some conditions for people receiving UC, such as preparing and searching for work or increasing your earnings. The benefit can be withdrawn or reduced (sanctioned) if those conditions are not met and there are some exceptions (e.g. long-term disability) [113] Value of qualifying benefit (including cap on value of benefits): Two-child cap: applicants on UC can receive up to an additional £3455 per year (for the 2024/2025 tax year) per child for a maximum of two children for those born after April 2017 [114]. Exemptions can apply in some circumstances, such as multiple births [113] Cap on housing benefit (“bedroom tax”): the amount of rent that working-age council or housing association tenants can claim a Housing Benefit for is capped if they are considered to be under-occupying their homes [115] (mitigated by Discretionary Housing Payments provided by the Scottish Government) [116] Graduation from benefits: Migration from current benefits to UC is underway. The “cliff-edge” withdrawal of the existing benefits (versus a gradual tapering of benefits) means that processing delays and missed notices can result in delays of full payments
Additional UK and Scottish government support	This refers to support provided by the UK government or the Scottish Government and includes childcare payments, support to mitigate disruptions caused by COVID-19, support for cost of living and expansion of early learning
Scotland Act 2016	The Scotland Act 2016 gave the Scottish Parliament responsibility for social security expenditure [16]. Social Security Scotland was created to implement the devolved powers. This agency is responsible for delivering the Five Family Payments, including the SCP
Child Poverty (Scotland) Act 2017	The Child Poverty (Scotland) Act 2017 commits the Scottish Government to reducing relative child poverty to below 10% by 2030/2031. Relative child poverty, which is the percentage of children living in households with equivalized net incomes of less than 60% UK median household income after housing costs, is estimated at 24% for the period 2020–23 [117]

*Amounts accurate as of May 2024. *DWP* Department for Work and Pensions, *HMRC* His Majesty’s Revenue and Customs, *UC* universal credit, *SCP* Scottish Child Payment

### Prioritizing outcomes and understanding data requirements

**Fig. 4:**
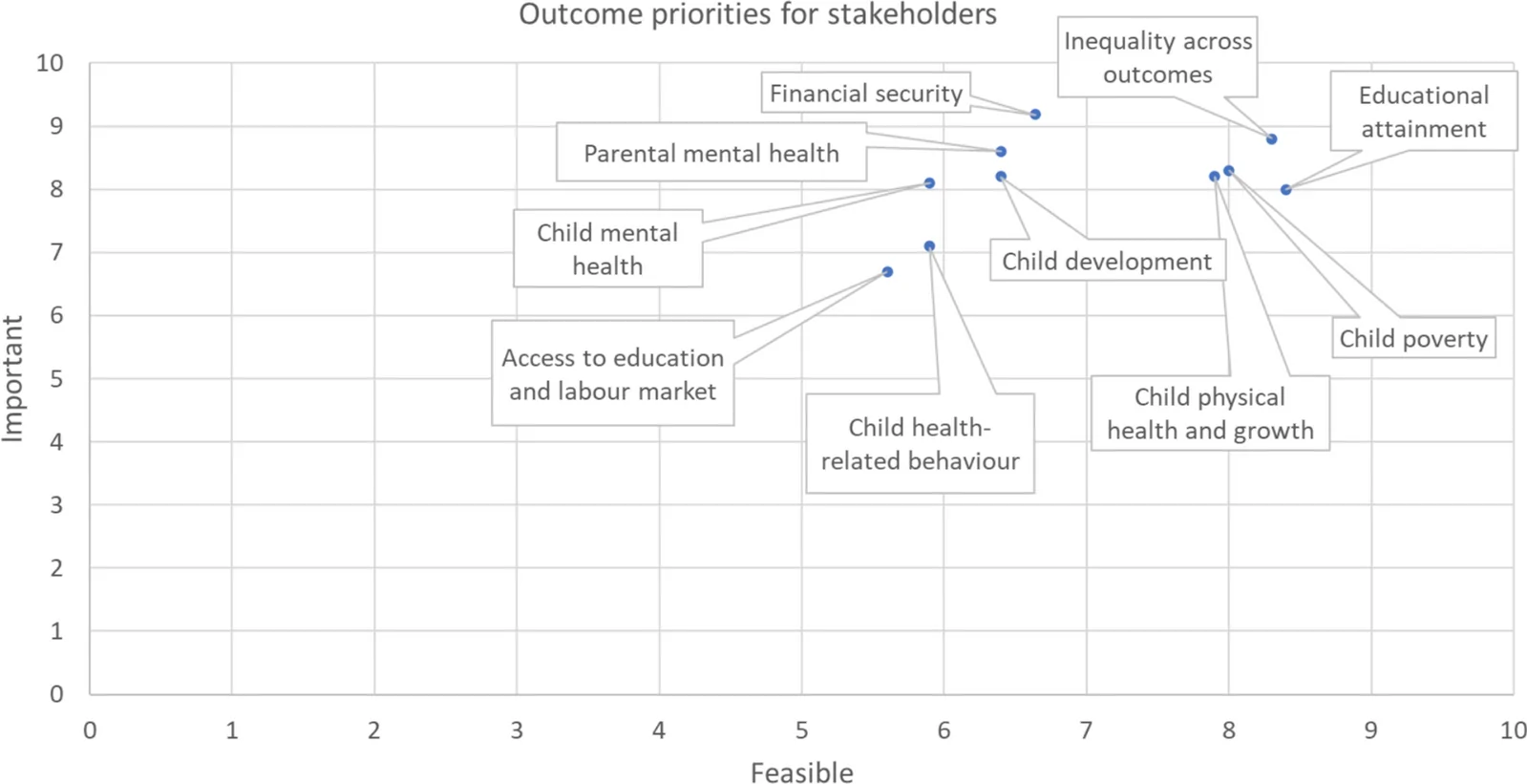
Stakeholder-ranked priorities for future evaluations, by combined importance (*y*-axis) and feasibility (*x*-axis) score based on Mentimeter voting from 1–10 (from least to most important and feasible)

Reflecting the discussions from workshop 1, outcomes related to “inequalities” between key groups of interest, particularly priority families, and the wider population received the highest combined score for importance and feasibility in the stakeholder vote (see Fig. 4). Whilst child poverty was also highly rated, stakeholders acknowledged that existing evaluations already address this outcome, a position later reaffirmed during feedback on the evaluation design options. The remaining priority outcomes were primarily related to the health and wellbeing of both parents and children, considered important for assessing whether children are receiving the best possible start in life, including child development and parental mental health.

#### Data requirements

There was strong consensus amongst stakeholders that future evaluations should prioritize quantifying the impact of the SCP on outcomes for children and families. After discussing a range of data sources, stakeholders concluded that, where possible, administrative data should be used to measure each outcome. They agreed that routinely collected data include relevant measures for many outcomes of interest (summarized in Table 3). For example, child development could be measured using health visitor data [59], and mental and physical health using hospital and prescription data [6]. Stakeholders also agreed that administrative datasets, which are comprehensively collected in Scotland and have population-wide coverage, would have more adequate power, and because of the population-wide coverage, also be less biased than many of the surveys discussed. This includes the Scottish Health Survey, which has a sample size of around 2000 children that is likely to be too small to detect SCP effects [60]. We also discussed the Growing Up in Scotland cohort, but as they were born in the early-to-mid 2000s, they were too old when the SCP was introduced [52, 61, 62]. Primary data collection was also ruled out, as pre- and post-intervention comparisons would have required prospective planning, whilst the multiple pre- and post-intervention data points that administrative data can provide means that key sources of bias, such as those introduced by pre-exposure trends, can be measured and accounted for [62]. Moreover, collecting primary data was considered less cost-efficient than using administrative data, as it requires substantial resources and often fails to overcome biases resulting from self-reporting and participation [63]. It was also unlikely to add value to the ongoing survey work led by Social Security Scotland [64].

**Table 3:** Possible data sources and measures for outcomes

Outcomes	Sources	Example measures
Household finances		
Financial security	FRS	Amount in savings; food security (households have sufficient food to facilitate an active and healthy lifestyle); household food bank usage
Child-related spending	None identified	Spending on materials to support learning; essential needs such as nappies, toys, books, treats; social and leisure opportunities
Child development		
Participation in social and educational opportunities	CHSP-PS; CHSP-S	Attends childcare (e.g. nursery, playgroup, registered childminder, none)
Child development before school	CHSP-PS; CHSP-S	Speech, language and communication; gross motor; fine motor; personal/social; emotional/behavioural; vision and hearing; problem-solving
Educational attainment	Attendance; SPC; SQA; CfE	Overall absences; absences by reason; exclusions for challenging or disruptive behaviour; reason support is needed for pupils with additional special needs; performing at level (numeracy, literacy, reading, writing); highest level of educational attainment
Health and wellbeing		
Parental mental health	SMR01; SMR04; PIS	Transferred to psychiatric hospital from general hospital (continuous care); prescriptions for antipsychotics and related drugs; prescribing for antidepressants; prescriptions for hypnotics and anxiolytics; prescriptions for attention deficit hyperactivity disorder (ADHD); principal diagnosis
Parental health-related behaviours	CHSP-PS; CHSP-S; NDIP	Any breast feeding; primary carer current smoker; immunization schedule; child exposed to second hand smoke; complete coverage of universal primary and end infancy immunizations; toothbrushing twice a day; child’s age when weaning foods introduced; dental registration and/or attendance in the last 12 months; birth weight; preterm birth; tobacco exposure during pregnancy; alcohol/substance misuse during pregnancy
Parenting and familial relationships	None identified	–
Child physical health and growth	SMR01; A&E; NDIP	Hospital admissions (e.g. injuries or respiratory infections); diagnosis codes; procedures (e.g. dental procedures)
Child health-related behaviour	HLS; SHS	Schools meeting PE targets; physical activity levels, fruit and vegetable consumption
Child mental health	SMR01; SMR04; PIS	Prescriptions for antipsychotics and related drugs; prescribing for antidepressants; prescriptions for hypnotics and anxiolytics; prescriptions for attention deficit hyperactivity disorder (ADHD); principal diagnosis; status on admission
Social isolation and loneliness	SHS	Subjective loneliness
Population and systems level		
Inequality across outcomes for children	As elsewhere, specific to outcome	All relevant outcomes compared with individuals or households not exposed to the SCP
Child poverty	CILIF	Changes in relative and absolute poverty
Changes in position of main carers within households	None identified	–
Economic	Scottish Government	Gross domestic product; labour productivity
Access to education and labour market	SHeS linked with HLS	Initial leavers destination; follow-up leavers destination; returners to education
Access to social services	None identified	–
Demand for social services	CAHMNS	Waiting times
Entry into care	LAC; CHSP-PS; CHSP-S	Changes in number of children entering care at the local authority level

*A&E* Accident & Emergency, *Attendance* Attendance, Absence, and Exclusions Survey, *CAHMNS* child and adolescent mental health services, *CfE* Achievement of Curriculum for Excellence, *CHSP-PS* Child Health Surveillance Programme-Pre-school, *CHSP-S* Child Health Surveillance Programme-School, *CILIF* children in low income families local statistics, *FRS* Family Resources Survey, *HBAI* households below average income dataset, *HLS* School Healthy Living Survey, *LAC* children and young people: looked after children, *NDIP* National Dental Inspection Programme, *PIS* Prescribing Information System, *SCP* Scottish Child Payment, *SHeS* Scottish Health Survey, *SHS* Scottish Household Survey, *SLD* National Statistics School Leaver Destinations Survey, *SMR00* Outpatient Attendance, *SMR01* General/Acute Inpatients and Day Case, *SMR02* Maternity Inpatients and Day Case, *SMR04* Mental Health Inpatient and Day Case, *SPC* Scottish Pupil Census

Quantifying the SCP’s impact on child and parental health, as well as inequalities across the outcomes of interest, was considered important and complementary to the extensive existing qualitative research, including interviews with parents and guardians on barriers to uptake and SCP usage [44, 64, 65]. Estimating effectiveness would also help avoid duplicating ongoing qualitative research, including by the Child Poverty Action Group (CPAG), one of the stakeholder organizations, which is exploring barriers and facilitators to uptake [66]. The SCP’s impact is likely to vary by outcome and policy phase, and our ability to capture impact will depend on data quality, making it difficult to predict the exact magnitude. However, stakeholders expected the overall effect to be small, as is typical for many national policies that, though targeted at specific groups, are likely to influence population-wide outcomes [18].

### Developing evaluation design options

Whilst there was widespread agreement on the priority for future evaluations, stakeholders also recognized that the primary challenge in quantifying the impact of the SCP would be identifying exposed households on the basis of receipt or eligibility. The Department for Work and Pensions (DWP) controls household income and benefit data across the UK, but this is only made available to researchers at the small area level, limiting linkage to individual health records [24, 67]. The Children Living in Low Income Families (CiLIF) index provides estimates of child poverty, although this is also only available at the small area level. The potential for linking DWP household or individual data to health records was discussed and at the time of writing remains unresolved [24].

### Appraising evaluation design options

The four evaluation design options presented and appraised in this section reflect the approaches discussed in the stakeholder workshops: a simple before-and-after analysis, an interrupted time series (ITS), a controlled interrupted ITS and a regression discontinuity design (RDD) (see Table 4) [68].

**Table 4:** Evaluation design options for the Scottish Child Payment (SCP)

#	Study design	Population (SCP phase, range of child month and year of birth)	Exposure group (SCP phase, timing**); child age at period start	Comparison group (SCP phase, timing**); child age at period start	Examples of strengths and limitations
0	Simple pre-/post-comparison	Mother–child pairs in Scotland likely eligible* for phase 3 and 4, *only* (born October 2008–August 2015)	After SCP exposure (*only* phases 3 and 4, November 2022–December 2024); 7 years and 3 months to 14 years and 1 month	Before SCP exposure (phases 1 and 2; February 2021–October 2022); 5 years 6 months to 12 years 4 months	Easiest to conduct Does not account for many key sources of bias (e.g. trends of worsening health across the population, influence of co-interventions) Using proxy measures (e.g. SIMD, NS-SEC) to identify pairs likely eligible and exposed to phase 3 may impact accuracy in identifying population
1	ITS	Mother–child pairs in Scotland likely eligible* for phases 3 and 4, *at minimum* (born October 2008–September 2016)	After SCP exposure (*at least* phases 3 and 4; November 2022–December 2024); 6 years, 2 months to 14 years, 1 month	Counterfactual scenario: exposed group had SCP not been introduced (on the basis of pre-exposure trends)	Accounts for pre-intervention trends, reducing potential bias Does not account for influence of co-interventions on population trends Potential spillover effect from earlier phases, that is, children < 6 years in phase 1 may skew the overall effect of phase 3 on everyone < 16 years Using proxy measures (e.g. SIMD, NS-SEC) to identify pairs likely eligible and exposed to phase 3 may impact accuracy in identifying population
2	Controlled ITS	Mother–child pairs in the UK likely eligible for phase 3 and 4*, *at minimum* (born October 2008–September 2016)	After SCP exposure in Scotland (at least phases 3 and 4, November 2022–December 2024); 6 years, 2 months to 14 years, 1 month	1. Same as ITS (counterfactual scenario using pre-exposure trends); 2. Children in Northern England and Wales likely eligible if SCP were available, for example, UC recipients (at least phases 3 and 4, November 2022–December 2024); 6 years, 2 months to 14 years, 1 month	Accounts for pre-intervention trends, reducing potential bias Control group helps address co-intervention confounding, though there remains a risk of bias if co-interventions differentially impact on Scotland versus the control area Potential spillover effect from earlier phases, that is, children < 6 years in phase 1 may skew the overall effect of phases 3 and phase 4 on everyone < 16 years Using proxy measures (e.g. SIMD, NS-SEC) to identify pairs likely eligible and exposed to phases 3 and 4 may impact accuracy in identifying population Risk of bias if the measures used to identify potentially eligible groups vary between the Scotland and the control area
3	RDD	Mother–child pairs in Scotland likely eligible for phase 3 *only* of children 2 years above/below age threshold (children born between January 2006–September 2008)	Children young enough to be eligible for SCP (phase 3, November 2022–March 2024); 14 years 2 months to 15 years, 5 months at the start of phase	Children in Scotland just too old for SCP eligibility SCP for non-eligible children (phase 3, November 2022–March 2024); 15 years 6 months to 16 years 10 months	Strong design that can account for time-varying confounders and co-interventions Potential spillover effect from earlier phases, that is, children < 6 years in phase 1 and phase 2 may skew the overall effect of phase 3 on everyone < 16 years Might not be relevant to mother–child pairs of children not in age range Smaller sample size than other designs (due to need to restrict to ages around eligibility threshold)

*#* evaluation option number, *SCP* Scottish Child Payment, *UC* universal credit, *ITS* interrupted time series, *RDD* regression discontinuity design, *SIMD* Scottish Index of Multiple Deprivation, *NS-SeC* National Statistics Socio-economic classification. *Mother–child pairs likely eligible for and exposed to the SCP, identified using proxy measures (e.g. SIMD, NS-SEC); **exposed for at least 6 months

**Fig. 5:**
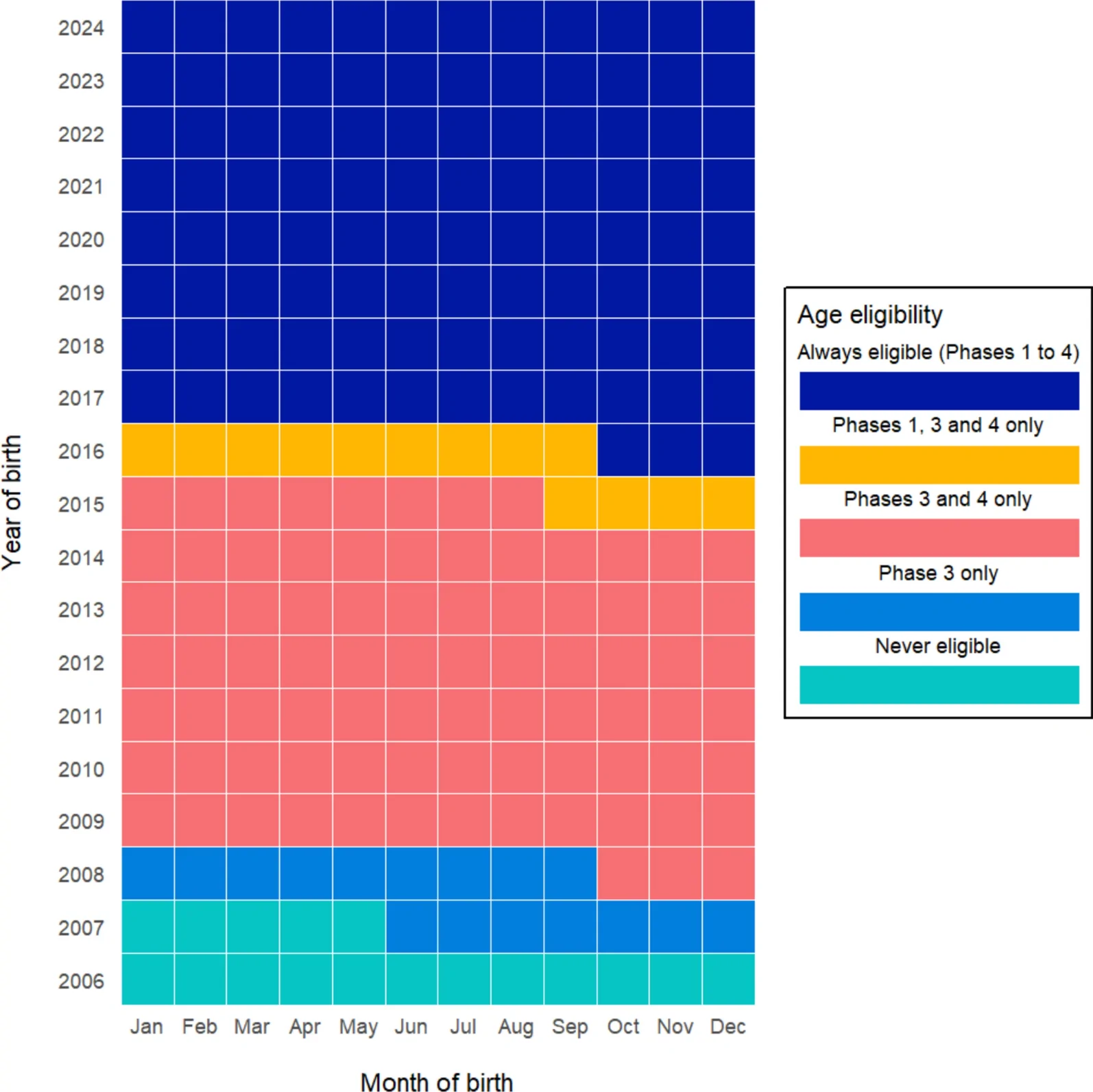
Eligibility for the Scottish Child Payment phases by year and month of birth from January 2006 to December 2024 (covering phases 1–4). A child is considered “eligible” for a phase if they are (or would be) under the age limit for at least 6 months during the phase, regardless of whether they were born when the phase started

The proposed study populations for all options are defined in terms of the child’s month of birth and their age at the time of the SCP phase from January 2006 to December 2024 (see Fig. 5). For simplicity, the four evaluation options described here have been tailored to phases 3 and/or 4 of the SCP, the most recent iteration of the policy. We anticipate the features of these options being relevant for other phases and a variety of outcomes, both of which should be further explored when developing an evaluation protocol. The study population for all the options, and where relevant, exposure to the SCP is approximated by combining multiple proxy measures from routinely collected data, such as area-level deprivation and/or child poverty (e.g. CiLIF) and individual and household demographic information (e.g. parent social class, recorded on birth certificates).

#### Option 0: pre-/post-design

Option 0 is a pre-/post-design considering the impacts of the SCP on mother–child pairs exposed to phases 3 and 4 for at least 6 months (orange shading in Fig. 5). This includes children born between October 2008 and August 2015, who were aged 7 years and 3 months to 14 years and 1 month at the start of phase 3 (November 2022). For simplicity, post-exposure outcomes are measured from November 2022 to December 2024 and compared with pre-exposure outcomes from February 2021 to October 2022, when these children were generally too old to be eligible under phases 1 and 2. A simple pre-/post-design does not account for factors that could bias outcomes before or after the start of phase 3 and 4, many of which were discussed in the stakeholder workshops, such as the impact of COVID-19 disruption on outcomes or changes in other qualifying benefits (see Table 2).

#### Option 1: interrupted time series

Option 1 uses an ITS design which compares outcomes after the intervention with a hypothetical, counterfactual scenario where pre-intervention trends continued [69]. The study population is defined as mother–child pairs exposed to *at least* phases 3 and 4 with children born between October 2008–September 2016 (orange and yellow shading in Fig. 5). As in option 0, the pre-exposure time is February 2021–October 2022. This means that some children in the option 1 study population will have received the SCP during phase 1 (those born between September 2015 and September 2016), whilst others will not have (those born between October 2008 and August 2015), as they were over 6 years old during phase 1 and therefore ineligible. The pre-exposure trends will differ for children depending on whether they were exposed only to phases 3 and 4 or to phase 1, 3 and 4, which can be accounted for in the analytical approach [70]. The difference between the study populations in option 0 (only exposed to phases 3 and 4) and option 1 (exposed to phases 3 and 4, with some also exposed to phase 1) underscores the importance of considering both the SCP phases and children’s birth month when defining the population.

#### Option 2: controlled interrupted time series

Option 2 is a controlled ITS which compares changes in outcomes of mother–child pairs in Scotland receiving the SCP before and after *at least* phase 3 and 4 with both a counterfactual scenario and a control group. The clearly defined administrative jurisdiction of the SCP, which is limited to Scottish residents, and its eligibility criteria based on United-Kingdom-wide benefit receipt (e.g. universal credit) make it particularly suitable for cross-country comparisons [71]. Including a control group that is similar to the treatment group – except in exposure to the intervention – can help distinguish the effects of the SCP from other events occurring at the same time that may also influence the outcomes, such as expanded UK Government support for the cost-of-living crisis (see Table 2) [62, 71, 72]. This could include selecting control group(s) from comparable areas in the rest of the United Kingdom, likely in Northern England and potentially Wales, where contextual trends are similar but the SCP has not been implemented [25]. Importantly, selecting controls from the rest of the United Kingdom requires in-depth assessment of their appropriateness, such as differences in income support policies and preexisting trends, as well as the suitability of existing datasets (e.g. Born and Bred in (BaBi) Network in Bradford [73], Secure Anonymised Information Linkage (SAIL) Databank in Wales [74]).

#### Option 3: regression discontinuity design

A RDD can be used when eligibility for a programme is determined by a continuous variable (known as a “forcing variable”) with a fixed threshold, where those within a narrow band either side of the threshold are comparable in all factors other than their eligibility for the intervention [57]. Option 3 applies an RDD to exploit the change in age eligibility for phase 3 of the SCP. For simplicity, we define the exposure group as children with at least 6 months of follow-up, setting the lower age limit to those born in January 2006. This results in a control group of mother–child pairs whose children just missed out on the SCP because they were slightly too old (born January 2006 to May 2007), but otherwise similar to the exposed group (born June 2007 to September 2008). In other words, this option compares children aged 14 years, 2 months to 15 years, 5 months with those aged 15 years, 6 months to 16 years, 10 months (blue shading versus green shading, respectively, in Fig. 5). This is a simplified illustrative example and in practice, key decisions such as treatment definition (e.g. minimum duration of SCP exposure) and the margin around the forcing variable that will be included in the study should be considered during the development of the study protocol.

## Discussion

Through close collaborations with stakeholders from government, the third sector and academia, this EA found a strong interest in estimating the effects of the SCP on child development and child and parental health and wellbeing and inequalities in these outcomes. Over several workshops, stakeholders refined an existing theory of change, prioritized outcomes and reviewed available data. There was strong consensus around the activities of the SCP, further indication that more work around understanding the policy is not needed [41, 75]. Stakeholders agreed that a quantitative evaluation would complement existing and ongoing qualitative research, which remains essential for designing and interpreting impact evaluation results. Stakeholders agreed that the outcomes of interest should be measured using linked administrative data and evaluated with natural experimental methods [41, 75]. There was also considerable interest in assessing effects on inequalities, including by the Scottish Government’s six priority family groups, though data limitations could restrict their precise identification.

### Key findings

#### Challenges in identifying exposure using administrative data

Administrative data were deemed to be best suited to quantitative evaluation; however, the existing barriers that limit cross-sectorial linkage make identifying families exposed to the SCP an important challenge commonly faced in policy evaluations [76]. Stakeholders therefore concluded that exposure to the SCP would likely need to be estimated using eligibility indicators recorded in administrative data, rather than direct identification [77]. For instance, parental occupation (recorded on birth certificates) could be combined with area-level data on the proportion of the population receiving SCP to estimate the likelihood of eligibility [53, 78]. These proxy measures may not completely predict SCP exposure [29, 79–81]. An alternative approach is to stratify age-eligible children (under 6 years) by area-level deprivation and test whether SCP-relevant outcomes differ across these groups. This approach has been used in evaluations of nationwide, area-based interventions, such as Sure Start centres [76]. However, the household-level nature of SCP payments creates challenges (e.g. estimating the total payments to households with multiple age-eligible and age-ineligible children) that simple area stratification cannot capture.

#### Challenges in identifying outcomes using administrative data

Administrative data provide valuable opportunities for evaluating policy impacts due to their large sample sizes and population coverage, but our stakeholder discussions highlighted key methodological challenges that future evaluations of the SCP must consider [52]. Researchers have limited control over the choice of indicators, and those available might not always be the most appropriate for measuring the outcomes of interest (e.g. responsive to the change in policy) [61]. Whilst we were able to identify measures for most outcomes in the refined theory of change, key outcomes such as improved financial security could not be captured using existing indicators [82, 83]. Data quality issues, including missing data, might further constrain the ability to detect subtle policy impacts [52, 84]. Data quality will vary by dataset, but Scotland’s administrative data typically offer high population coverage [6, 85]. This is particularly true for child and maternal health records, where, apart from ethnicity, studies report > 90% completeness for key variables such as birthweight, maternal smoking and socioeconomic indicators [53, 86]. We also discussed the challenges in interpreting some indicators; for example, a reduction in waiting times, measured from initial referral times to treatment, might reflect improved access to treatment, but could also indicate fewer referrals [52, 83]. The evaluation design options we proposed will need to be tailored to specific outcomes, and future evaluations of the SCP using administrative data are likely to face similar considerations to those we encountered when focussing on child and maternal health and wellbeing.

### Findings in relation to other studies

As noted, a range of qualitative work is already underway on the SCP, and a quantitative estimate would usefully complement this. Some of the ongoing research includes a project led by CPAG exploring the barriers and facilitators to accessing the SCP [66], as well as work by Social Security Scotland examining families’ experiences with all Five Family Payments such as how families navigate the application process, their interactions with staff and the timeliness and reliability of payments [64]. There is also a mixed-methods study comparing experiences of devolved social security benefits, including the SCP, for each nation across the United Kingdom [87].

Whilst many aspects of the evaluations we propose for estimating the impact of the SCP are specific to its implementation, such as a phased rollout, there are important lessons to draw from evaluations of other policies aimed at improving child and maternal health and wellbeing. For example, an evaluability assessment of the Family Nurse Partnership (FNP), a home visiting programme for first-time teenage mothers, similarly recommended the use of natural experiment methods harnessing administrative data [88]. The FNP evaluation examined many of the same outcomes we proposed for the SCP, using similar routinely collected datasets, thereby supporting the feasibility of our approach [52]. Both the FNP evaluation and recent Sure Start evaluation noted that hospitalizations might reflect either positive care-seeking behaviour or falls in disease incidence, highlighting the need for explicit acknowledgement to avoid misinterpretation [76, 89]. Stakeholders agreed that despite this ambiguity, these outcomes remain relevant for service planning purposes, noting that qualitative research and triangulation with other health outcomes may help contextualize any observed changes in hospitalization rates [90].

Other evaluations helped shape our evaluation design options, including that of the Health in Pregnancy Grant, a £190 lump sum provided to all pregnant mothers in the United Kingdom who were at least 25 weeks pregnant [53]. A controlled ITS evaluating the grant found no effect on birthweight, whilst a RDD using child birthdate as the forcing variable detected improvements in birthweight and reductions in low birthweight [91]. These differences were likely due to contemporaneous factors, such as the 2008–2009 financial crisis, which may have biased the ITS results downward [91]. The different approaches to evaluating the Health in Pregnancy grant underscored the risk of time-varying confounding in an ITS and the need for a large sample size in a RDD. This example also informed the development of the RDD (option 3) for evaluating the SCP, which also uses child birthdate as the forcing variable.

### Strengths and limitations

A key strength of our EA was engaging with stakeholders early, which fosters strong relationships and ensures that findings are relevant for decision-making [92]. Additionally, we established clear boundaries for roles and responsibilities, helping align the EA activities with stakeholders’ information needs and supporting findings that can be used for future evaluations [34, 40]. Stakeholder engagement, including written feedback on the four evaluation options, made these final options more relevant and credible [34, 65]. Moreover, though an EA should be tailored to the policy or programme being evaluated, some aspects of the findings from this EA might be relevant for other policies in Scotland, including the evaluation designs and secondary data sources identified.

Our EA built upon an existing SCP theory of change. Refining rather than reworking the framework helped manage the duration of the EA, avoid duplication and ensure sufficient detail [42, 47, 84]. This approach may have limited the identification of unexpected or harmful impacts, or underemphasized outcomes in other domains such as education. Cost-effectiveness was beyond the scope of our EA, which aimed to understand the feasibility and priorities for a robust evaluation of the SCP. It also did not emerge in our stakeholder discussions, likely reflecting interest in first establishing the SCP’s effectiveness, as an economic evaluation would provide important complementary information for decision-making. We sought to minimize this limitation by drawing on insights from the public engagement activities conducted as part of the broader NIHR project, which included discussions on unexpected or harmful outcomes. Additionally, we reviewed relevant literature prior to the workshops to identify a wide range of outcomes, as well as by bringing together diverse perspectives through our stakeholder group.

### Research and policy implications

#### Improving access to benefits data and linkage with health data

Given the ongoing discussions throughout the EA about the challenges of using administrative data to identify SCP exposure at the household level, researchers should continue to advocate for access to benefits data and other non-health administrative records, as well as facilitating linkage to health records [67]. This would strengthen evaluations of both the SCP as well as other social security policies, as relying on eligibility indicators in administrative data is likely to introduce measurement error and lead to underestimation of effects [93]. Moreover, improving direct identification can have wider benefits for effective policymaking, including better identification and take-up of benefits, enhanced policy modelling and support for automation efforts [24]. Progress will depend on data sharing agreements between relevant agencies, which researchers [67, 94] and the UK Government are currently working on [95]. Improved access to benefits data would not only strengthen the exposure measurement to the SCP, but also facilitate an analysis of inequalities, for example, by using disability benefits to identify one of the Scottish Government priority groups.

#### Tailoring the evaluation design to the research question

Stakeholders expressed strong interest in natural experiment studies, and we proposed four potential evaluation designs. Each option is feasible and offers difference strengths and limitations; thus the most appropriate design will depend on the specific research questions. One of the main challenges raised during the workshops was how to isolate the effects of the SCP from co-occurring contextual factors. A controlled ITS can help disentangle the effects of contemporaneous factors impacting both the exposed and control groups. Although other UK nations were proposed as potential control groups, a cross-country evaluation requires careful consideration, including whether meaningful differences from Scotland would be expected if other nations have their own poverty reduction programmes [96, 97], as well as the resources needed to access and align data from different countries. Moreover, the context has shifted since this EA was conducted. UK-wide developments, such as policy changes following the July 2024 general election and the uneven impacts of COVID-19 across the UK, now need to be taken into account [98, 99].

In addition to isolating policy effects, the phased rollout of the SCP should also factor into considerations regarding the most suitable evaluation design. For example, a RDD comparing children just below and above the age cutoffs could control for Scotland-specific co-interventions, such as discretionary housing payments. Whilst a RDD is methodologically robust, its application is limited to children near the SCP thresholds (ages 6 and 16 years), which may limit the relevance of the evaluation for decision-makers. Researchers must assess whether this design would yield a sufficiently large sample to ensure co-interventions and other confounders are evenly distributed [57]. Careful definition of the bandwidth around the cutoff is critical, as it determines the age range of treatment and control groups and should be tested through sensitivity analyses. Findings should be triangulated across multiple design options with complementary strengths and weaknesses, each subject to different sources and directions of bias, whilst negative controls provide additional robustness [90, 100, 101]. Tailoring the evaluation design to the specific research question should be explored during the development of the analysis protocol [65, 102].

## Conclusions

The SCP is an important part of the poverty-reduction strategy in Scotland, and a robust evaluation of its quantitative impacts is essential. Future evaluations must consider the evolving phases of the SCP, including changes in eligibility and value. With health and health inequalities considered the priority outcomes, administrative data should be used wherever possible due to its broad coverage and large sample size. However, challenges persist, including the identification of exposed households, the measurement of relevant outcomes and the interpretation of results given the complex analytic approaches required. To address these issues and support rigorous policy evaluations, ongoing investment in data linkage infrastructure is needed, particularly between health and non-health administrative datasets.

## Supplementary Information

Supplementary material 1.

## Data Availability

No datasets were generated or analysed during the current study.
