# Supplementary material for: Evaluability assessment of a targeted national anti-child poverty policy: option appraisal for evaluating the Scottish Child Payment
Source: Health Res Policy Syst. 2026 Jun 17;24:86. doi: 10.1186/s12961-026-01489-y (PMC13647560; doi:10.1186/s12961-026-01489-y)
Supplement: Supplementary file 1 [file 12961_2026_1489_MOESM1_ESM.docx]

Supplementary information for evaluability assessment of the Scottish Child Payment

# Supplementary table 1

*Supplementary table 1 Stakeholder organisations consulted about a potential Scottish Child Payment (SCP) evaluation*

| **Organisation** | **Description of organisation** |
| --- | --- |
| Public Health Scotland | Public Health Scotland is an NHS Board and Scotland’s national public health body (https://publichealthscotland.scot). PHS leads work across Scotland to prevent disease and promote health and wellbeing with the overarching aim of improving life expectancy and reducing health inequalities amongst the Scottish population. PHS works across a range of areas to inform decision making and promote evidence-based policy through the data, research, and intelligence it produces in order to make the biggest impact on improving health in Scotland. |
| Scottish Government | The Scottish Government is the devolved government for Scotland; it runs the country in relation to matters that are devolved from Westminster (https://www.gov.scot). These include: the economy, education, health, justice, rural affairs, housing, environment, equal opportunities, consumer advocacy and advice, transport, and taxation. |
| Child Poverty Action Group | Child Poverty Action Group is the UK’s leading charity working to prevent child poverty (https://cpag.org.uk). Their primary objectives are to campaign to strengthen public commitment to end child poverty; ensure government adopt evidence-based policy solutions to child poverty; and use their expertise to maximise family incomes and reduce child poverty. |
| Nesta (formerly NESTA, National Endowment for Science, Technology and the Arts) | Nesta are the UK’s innovation agency for social good. Nesta work to support, encourage, and inspire innovation that benefits society by designing, testing, and scaling new solutions to society’s biggest problems (https://www.nesta.org.uk). Their current strategy focuses on three innovation missions: a fairer start for every child; a healthy life for all, and a sustainable future where the economy works better for people and the planet. |
| Research Data Scotland | Research Data Scotland is a not-for-profit charitable organisation created and funded by the Scottish Government with the aim of improving access to Scottish public sector data for research that benefits the public (https://www.researchdata.scot). RDS is a partnership between the Scottish Government, Scotland’s leading universities, and public bodies, who are working to simplify access to public sector data through providing a single point of contact for safe, secure, and effective access to secure datasets. |
| Joseph Rowntree Foundation | The Joseph Rowntree Foundation is a charity in the UK focused on addressing poverty in the UK (https://www.jrf.org.uk). It carries out work including policy development, advocacy and campaigns, and research on causes and solutions to poverty. |

CPAG = Child Poverty Action Group; JRF = Joseph Rowntree Foundation; RDS = Research Data Scotland; NHS = National Health Service; PHS = Public Health Scotland.

# Supplementary table 2

Supplementary table 2 Description of the inputs of the Scottish Child Payment (SCP).

| **Inputs** | **Description** |
| --- | --- |
| **Governments** | |
| Funding by Scottish Government | This includes funding which has been made available since social security was devolved under the Scotland Act 2016. |
| **Agencies** | |
| Staff time and resources at Social Security Scotland | The time and resources required by staff at Social Security Scotland to, among other things, process applications, respond to applicant queries, and support people in applying. Considered to be a key and important input. |
| Access to information to process applications. | Key information required to process applications includes confirmation that applicants receive qualifying benefits (e.g., UC). |
| Resources to promote the SCP | The ways in which parents heard about SCP are monitored via the client survey. However, it is too soon to know how groups who only recently became eligible (i.e. older children) are being reached and they may require different approaches. There are no specific plans to develop auto-enrolment, which further suggests that resources to promote the payment remain important. |
| **Community** | |
| Resources to support engagement with the SCP | These are the community organisations and partners that provide the information and support which can help people engage and apply for the SCP. This includes Deep-End practitioners, the Face up to Child Poverty campaign in school, and child development activities in local authorities. |
| **Applicants** | |
| Time and evidence needed to apply | Recipients of the SCP will be automatically re-enrolled if they become ineligible and then eligible within 12 weeks (e.g., sanctioned by UC), meaning there is no need to reapply in those circumstances. However, in all other circumstances an application is required to receive the benefit. |

UC = Universal credit; SCP = Scottish Child Payment.

# Supplementary table 3

Supplementary table 3 Description of Scottish Child Payment (SCP) activities.

| Activities | Description and notes |
| --- | --- |
| Cash payment of £26.70 per week per child aged under 16 years to parents or guardians on qualifying benefits*. | Payments are made every four weeks, in arrears, into the bank account of the responsible parent or guardian. |
| Automatic enrolment into Best Start Grant Early Learning and School Age Payments. | Children who receive SCP automatically receive payments for Best Start Grant Early Learning and School Age when they become eligible. There are currently no plans for auto-enrolling families receiving SCP into other payments. |

*Amounts accurate as of May 2024; SCP=Scottish Child Payment.
